# UNDERSTANDING THE PREDICTIVE FACTORS THAT INFLUENCE THE PERCEIVED BURDEN OF DEMENTIA CAREGIVERS

**DOI:** 10.1093/geroni/igac059.2204

**Published:** 2022-12-20

**Authors:** Kaitlyn Lucas, Katherine Judge

**Affiliations:** Cleveland State University, Westlake, Ohio, United States; Cleveland State University, Cleveland, Ohio, United States

## Abstract

Burden is a significant negative outcome for caregivers of individuals with dementia. Guided by the Stress Process Model, the following study examined the impact of several novel constructs on burden including: compassion for others, family functioning, coping styles, and positive and negative affect. Data were gathered through Amazon Mechanical Turk from 102 dementia caregivers. Participants were 62% female, 72% Caucasian with a mean age of 39.5 years old. Burden was significantly related to positive affect (r =-.31, p =.01), negative affect (r =.56, p =<.01), family functioning (r =.43, p <.01), and dysfunctional coping (r =.37, p <.01). Compassion for others was not correlated with burden. Multiple regression analysis results found the set of predictors accounted for 65.0% of the total variance in burden (F(5,96) = 14.17, p < .01, R2= .65), with positive affect (β = -.26, p = .01), negative affect (β = .19, p= .05), and dysfunctional coping (β = .31, p= .01) predicting significant and unique variance. Results from mediation analyses indicated dysfunctional coping mediated the relationship between positive affect and burden (effect= -.14 95% bootstrap CI= -.26, -.04) as well as the relationship between negative affect and burden (effect= .24, 95% bootstrap CI= .07, .48). A mediating relationship between dysfunctional coping and family functioning and burden was not supported. Discussion will highlight the implications of the study findings in developing innovative caregiver programs aimed at reducing dysfunctional coping and the broader theoretical implications of the role of affect in family caregivers of individuals with dementia.

